# Cell factories converting lactate and acetate to butyrate: *Clostridium butyricum* and microbial communities from dark fermentation bioreactors

**DOI:** 10.1186/s12934-019-1085-1

**Published:** 2019-02-13

**Authors:** Anna Detman, Damian Mielecki, Aleksandra Chojnacka, Agnieszka Salamon, Mieczysław K. Błaszczyk, Anna Sikora

**Affiliations:** 1 Institute of Biochemistry and Biophysics – Polish Academy of Sciences, Pawińskiego 5a, 02-106 Warsaw, Poland; 2 Institute of Agricultural and Food Biotechnology, Rakowiecka 36, 02-532 Warsaw, Poland; 3 Faculty of Agriculture and Biology, Warsaw University of Life Sciences, Nowoursynowska 159, 02-776 Warsaw, Poland

**Keywords:** Lactate, Acetate, Butyrate, *Clostridium butyricum*, Microbial communities, Fermentation, Etf complexes, Phylogenetic analysis, Metabolic pathways

## Abstract

**Background:**

Interactions between microorganisms during specific steps of anaerobic digestion determine metabolic pathways in bioreactors and consequently the efficiency of fermentation processes. This study focuses on conversion of lactate and acetate to butyrate by bacteria of dark fermentation. The recently recognized flavin-based electron bifurcation as a mode of energy coupling by anaerobes increases our knowledge of anaerobic lactate oxidation and butyrate formation.

**Results:**

Microbial communities from dark fermentation bioreactors or pure culture of *Clostridium butyricum* are able to convert lactate and acetate to butyrate in batch experiments. The ability of *C. butyricum* to transform lactate and acetate to butyrate was shown for the first time, with ethanol identified as an additional end product of this process. A search for genes encoding EtfAB complexes and their gene neighbourhood in *C. butyricum* and other bacteria capable of lactate and acetate conversion to butyrate as well as butyrate-producers only and the lactate oxidiser *Acetobacterium woodii*, revealed that the Etf complexes involved in (i) lactate oxidation and (ii) butyrate synthesis, form separate clusters. There is a more extent similarity between Etf subunits that are involved in lactate oxidation in various species (e.g. *A. woodii* and *C. butyricum*) than between the different *etf* gene products within the same species of butyrate producers. A scheme for the metabolic pathway of lactate and acetate transformation to butyrate in *C. butyricum* was constructed.

**Conclusions:**

Studies on the conversion of lactate and acetate to butyrate by microbial communities from dark fermentation bioreactors or *Clostridium butyricum* suggest that a phenomenon analogous to cross-feeding of lactate in gastrointestinal tract also occurs in hydrogen-yielding reactors. A scheme of lactate and acetate transformation pathway is proposed, based on the example of *C. butyricum*, which employs flavin-based electron bifurcation. This process utilizes electron-transferring flavoprotein (Etf) complexes specific for (i) lactate oxidation and (ii) butyrate formation. Phylogenetic analysis revealed that such complexes are encoded in the genomes of other bacteria capable of lactate and acetate conversion to butyrate. These findings contribute significantly to our understanding of the metabolic pathways and symbiotic interactions between bacteria during the acidogenic step of anaerobic digestion.

## Background

The conversion of lactate and acetate to butyrate (cross-feeding of lactate) is a recognized nutritional interaction between lactate- and acetate-forming bacteria and butyrate producers. This process has been investigated in vitro using co-cultures of bacteria isolated from the human gut: *Bifidobacterium adolescentis* and bacteria related to *Eubacterium hallii* and *Anaerostipes caccae* [1–3].

Previously, we postulated that a phenomenon analogous to cross-feeding of lactate in the gastrointestinal tract also occurs in dark fermentation bioreactors, since the ability to produce butyrate from lactate and acetate seems to be shared by members of the genus *Clostridium* and other hydrogen-producing bacteria capable of butyric acid fermentation of carbohydrates [4]. In the absence of carbohydrates, *Clostridium acetobutylicum* strain P262 [5], *Butyribacterium methylotrophicum* [6], and *Clostridium diolis* [7] can utilize lactate and acetate, converting them to butyrate, carbon dioxide and hydrogen. The results of a number of studies indicate that the presence of lactic and acetic acids within fermentation substrates can stimulate biohydrogen production [7–11].

Etchebehere et al. [12] analysed the hydrogen-producing microbial communities in bioreactors with a low, medium or good performance by 454 pyrosequencing. The presence of lactic acid bacteria (LAB) was found to be correlated with low reactor performance. However, LAB were as abundant as *Clostridium* spp. in the most efficient bioreactors. These finding support our proposal that LAB perform an important function in hydrogen-yielding microbial communities as competitors or stimulators of hydrogen producers, and help to balance specific groups of bacteria in bioreactors [4].

Butyric acid fermentation is bacterial fermentation well-recognized for many saccharolytic species of *Clostridium*, e.g. *C. butyricum*. Biochemistry of butyrate formation is commonly accepted [13]. The key reactions of butyrate synthesis is formation of butyryl-CoA. It is endergonic ferredoxin reduction with NADH coupled to exergonic crotonyl-CoA reduction with NADH catalyzed by the butyryl CoA dehydrogenase/Etf complex (Bcd/EtfAB complex): 2NADH + Fd_ox_ + crotonyl-CoA → 2 NAD + Fd_red_ + butyryl-CoA. Biochemistry of the reaction was described for *Clostridium kluyveri* [14] and then confirmed in other *Firmicutes* [15, 16]. Genomes searches have revealed that genes encoding CoA dehydrogenase and Etf complex are commonly found in butyrate producing *Firmicutes* [17].

Flavin-based electron bifurcation has been recognized as a third mode of energy coupling in anaerobes (*Bacteria* and *Archaea*). It involves coupling exergonic and endergonic electron transfer reactions to generate a net exergonic reaction with minimal negative free energy change and maximal energy conservation [18, 19].

Flavin-based electron bifurcation has also explained biochemistry of lactate oxidation on the example of *Acetobacterium woodii*. The oxidation of lactate to pyruvate requires the activity of a FAD-dependent lactate dehydrogenase (LDH)/electron transferring flavoprotein (EtfAB) complex that catalyzes ferredoxin dependent reduction of NAD by lactate: 2 NAD + Fd_red_ + lactate → 2NADH + Fd_ox_ + pyruvate [20]. The genes for lactate utilization under anaerobic conditions are widespread in the domain *Bacteria* [20, 21].

According to our thesis the discovery of flavin-based electron bifurcation helps to increase our understanding of metabolic pathways of lactate and acetate transformation to butyrate and fermentation gases that were proposed previously [1, 5, 6].

Recent studies based on bioinformatic and structural analyses have revealed that Etf enzymes are phylogenetically diverse and widely distributed in the domains *Bacteria* and *Archaea*. They distinguished five distinct Etf groups named G1-G5. The Etfs involved in butyrate and lactate metabolism are bifurcating enzymes and belong exclusively to group G2. Furthermore, group 2 is divided into two subgroups. Subgroup G2A includes Etfs involved in butyrate metabolism, while subgroup G2B contains those involved in lactate metabolism [22].

The aim of the study was to confirm that bacteria of dark fermentation are able to convert lactate and acetate to butyrate and to propose an enzymatic machinery involved in this process. The transformation of lactate to butyrate was studied in batch experiments using media containing molasses supplemented with lactate and acetate, or a mixture of lactate and acetate without added carbohydrates, inoculated with samples of microbial communities from dark fermentation bioreactors [23] or a pure culture of *Clostridium butyricum* 2478. Phylogenetic analysis of the EtfAB complexes from *Firmicutes* species capable of lactate to butyrate transformation and *Acetobacterium woodii* as a lactate-oxidizer, was performed. An updated scheme of the metabolic pathway of lactate and acetate transformation is proposed.

## Methods

### Bacteria and media

The bacteria used in the tests of lactate and acetate to butyrate conversion were samples of microbial communities from dark fermentation bioreactors described previously [23] and *Clostridium butyricum* 2478 (DSMZ collection, DSM-2478).

The liquid growth medium was M9 [24] without glucose, supplemented with molasses at a concentration corresponding to 2% sucrose; sodium lactate (Chempur Poland) 7.41 g/L; sodium acetate (Chempur Poland) 7 g/L; and 0.2% yeast extract (BD Bioscences USA) in different combinations, summarized in Table 1. Molasses came from the Dobrzelin Sugar Factory (belonging to the Polish Sugar Company “Polski Cukier”). For the cultivation of *C. butyricum*, clostridial differential medium (CDA) (Sigma Aldrich) was also used. Starting pH of all media was 7.0. Since the M9 medium contains phosphates and possesses buffer properties no additional pH control was used.

**Table 1:** Tests on transformation of lactate and acetate to butyrate

Bacteria	M9 medium without glucose supplemented with	Number of experiments	Duration	pH of the culture before^a^			Density of the culture OD_600nm_ before						Non-gaseous fermentation products
				1st passage	2nd passage	End sampling	1st passage		2nd passage		End sampling		
							2nd day	3rd day	2nd day	3rd day	2nd day	3rd day	
Microbial community from a dark fermentation bioreactor	Molasses	3	9 days, 2 passages (every 3 days)	4.6–4.8	4.7–4.8	4.6–4.9	0.5–0.6	2.7–3.1	0.4–0.5	2.3–3.1	0.5–0.6	2.9–3.0	Figure 1a
	Molasses and sodium lactate	3		4.8–5.5	4.8–5.5	4.6–5.0	0.7–0.8	2.0–2.6	0.6–0.8	1.8–3.2	0.7–0.8	2.4–2.6	
	Molasses, sodium lactate and sodium acetate	2		4.7–5.0	4.6–5.1	4.7–4.8	0.5–0.7	1.5–3.1	0.5–0.6	1.6–2.3	0.5–0.6	2.5–2.6	
				6.5–6.7	6.7–6.8	6.5–6.7	0.2–0.3	0.9–1.0	0.2–0.3	0.8–1.0	0.2–0.3	0.8–1.0	Figure 1b

Bacteria	M9 medium without glucose supplemented with	Number of experiments	Duration	pH of the culture after			Density of the culture OD_600nm_ after						Non-gaseous fermentation products
				3 days	6 days	9 days	3 days		6 days		9 days		
*C. butyricum*	Sodium lactate, sodium acetate and yeast extract	3	9 days, no passage	6.8–6.9	6.8–7.0	6.8–7.1	~ 0.20		~ 0.6		~ 0.7		Figure 1c

^a^pH of the starting media was 7.0 in all cases

### Experimental set-up for the examination of lactate to butyrate transformation

All bacterial cultures were grown anaerobically in a Vinyl Anaerobic Chamber (Coy Laboratory Products, Inc.) without shaking at 30 °C. Bacterial growth was determined by OD_600 nm_ measurements.

Tests on the transformation of lactate and acetate to butyrate by the microbial communities from dark fermentation bioreactors were conducted in batch experiments in 250-ml Erlenmayer flasks for 9 days. At the start of each experiment a single granitic stone, acting as packing material in the packed-bed bioreactor [23, 25] and covered by bacterial biofilm, was placed in each flask as the inoculum and covered with 100 ml of growth medium. After 3 days of incubation the stone was transferred to a flask containing fresh medium (the first passage) for further growth. The procedure was repeated (the second passage). After another 3 days, the cultures were centrifuged and the supernatants analysed as described below.

To test the ability of *C. butyricum* to transform lactate and acetate to butyrate, tubes containing 15 ml of fresh M9 medium plus sodium acetate and sodium lactate without added carbohydrates and supplemented with yeast extract, were inoculated with *C. butyricum*. These cultures were incubated as described above for 9 days.

All experimental variants are summarized in Table 1. Composition of the selective media and lactate and acetate concentration were proposed after analysis of other studies [7–9].

### Analytical methods

The pH of the media and the cultures was measured using a standard pH meter (ELMETRON model CP-502, Poland). The concentration of carbohydrates in the molasses-containing media and culture supernatants was analyzed using high performance liquid chromatography (HPLC) with refractometric detection (Waters HPLC system: Waters 2695—Separations Module, Waters 2414—Refractive Index Detector, and 300 × 6.5 mm Sugar Pak column with guard column). Short-chain fatty acids were analyzed by HPLC with photometric detection (Waters HPLC system as above, Waters 2996—Photodiode Array Detector, and 300 × 7.8 mm Aminex HPX-87 H column with guard column). Ethanol was quantified by gas chromatography with flame-ionization detection (Hewlett Packard 6890, autosampler headspace—Hewlett Packard 7694E, polar 1.0-μm capillary column and flame ionization detector, FID). The HPLC conditions used for these analyses were as described previously [23, 25].

### Identification of *etfA/B* and neighbouring genes in *Clostridium butyricum* KNU-L09

Searches using tBLAST [26] were performed for the sequences of two chromosomes of *Clostridium butyricum* KNU-L09 (NCBI RefSeq: NZ_CP013252, NZ_CP013489) with a custom made database. The *etfA* (locus_tag: AWO_RS04415) and *etfB* (AWO_RS0441) genes of *Acetobacterium woodii* DSM 1030 (NC_016894) [20] were used as queries. Searches, data analysis and visualisations were performed with Geneious 10.2.4 [27]. The *etf* sequences from different clostridial species were also used as queries and they gave identical results.

Similar search, with *A. woodii* EtfA and EtfB and additionally with its l-lactate permease (AWO_RS04425), was performed for the genomes of *Roseburia intestinalis* L1-82 (TaxID: 536231, RefSeq: NZ_ABYJ00000000), *Eubacterium rectale* ATCC 33656 (TaxID: 515619, RefSeq: NC_012781), and *Faecalibacterium prausnitzii* A2165 (RefSeq: NZ_CP022479) [1, 28].

### Phylogenetic analysis of EtfA and EtfB proteins from the selected bacterial species

The EtfA and EtfB protein sequences were searched in the genome sequences of the following species: *Acetobacterium woodii* DSM1030 (TaxID: 931626, RefSeq: NC_016894), *Butyribacterium methylotrophicum* DSM3468 (TaxID: 1487, Assembly: ASM175369v1), *Clostridium acetobutylicum* ATCC 824 (TaxID: 272562, RefSeq: NC_003030, NC_001988), *Clostridium butyricum* KNU-L09 (TaxID: 1492, RefSeq: NZ_CP013252, NZ_CP013489), *Clostridium diolis* NJP7 (TaxID: 223919, Assembly: ASM217689v1), *Clostridium kluyveri* DSM555 (TaxID: 431943, RefSeq: NC_009706, NC_009466), *Megasphaera elsdenii* DSM20460 (TaxID: 1064535, RefSeq: NC_015873).

A custom BLAST database was prepared from the genome sequences of the above listed species using Geneious software [27], and tBLASTn [29] searches were performed with the *A. woodii* DSM 1030 EtfA (AWO_RS04415) and EtfB (AWO_RS04410), with default parameters (Matrix: Blosum62, Gap cost: 11, Gap extend: 1, Word size: 6).

The selected EtfA and EtfB proteins were subjected to phylogenetic analysis. The particular EtfA and EtfB proteins (locus tags in parenthesis) were named (Fig. 3) according to the genetic context in which they were found.

For EtfA: A_woodii_DSM1030_acyl-CoA (AWO_RS08105), A_woodii_DSM1030_GlcD_LldP (AWO_RS04415), B_methylotrophicum_DSM3468_acyl-CoA (BUME_07090), B_methylotrophicum_DSM3468_GlcD_1 (BUME_04260), B_methylotrophicum_DSM3468_GlcD_2 (BUME_04230), B_methylotrophicum_DSM3468_GlcD_LldP (BUME_24810), C_acetobutylicum_ATTC824_3-hydroxybutyryl-CoA (CA_C2709), C_acetobutylicum_ATTC824_GlcD (CA_C2543), C_butyricum_KNU-L09_3-hydroxybutyryl-CoA (ATN24_RS03165), C_butyricum_KNU-L09_GlcD (ATN24_RS03030), C_butyricum_KNU-L09_LldP_GlcD (ATN24_RS08885), C_diolis_NJP7_3-hydroxybutyryl-CoA (CCS79_RS24290), C_diolis_NJP7_GlcD (CCS79_RS18270), C_diolis_NJP7_LldP_GlcD (CCS79_RS09600), C_kluyveri_DSM555_3-hydroxybutyryl-CoA (CKL_RS02260), C_kluyveri_DSM555_GlcD (CKL_RS17115), M_elsdenii_DSM20460 (MELS_RS10255), and M_elsdenii_DSM20460_acyl-CoA (MELS_RS10960).

For EtfB: A_woodii_DSM1030_acyl-CoA (AWO_RS08100), A_woodii_DSM1030_GlcD_LldP (AWO_RS04410), B_methylotrophicum_DSM3468_acyl-CoA (BUME_07100), B_methylotrophicum_DSM3468_GlcD_1 (BUME_04270), B_methylotrophicum_DSM3468_GlcD_2 (BUME_04240), B_methylotrophicum_DSM3468_GlcD_LldP (BUME_24820), C_acetobutylicum_ATTC824_3-hydroxybutyryl-CoA (CA_C2710), C_acetobutylicum_ATTC824_GlcD (CA_C2544), C_butyricum_KNU-L09_3-hydroxybutyryl-CoA (ATN24_RS03160), C_butyricum_KNU-L09_GlcD (ATN24_RS03025), C_butyricum_KNU-L09_LldP_GlcD (ATN24_RS08880), C_diolis_NJP7_3-hydroxybutyryl-CoA (CCS79_RS24285), C_diolis_NJP7_GlcD (CCS79_RS18265), C_diolis_NJP7_LldP_GlcD (CCS79_RS09595), C_kluyveri_DSM555_3-hydroxybutyryl-CoA (CKL_RS02255), C_kluyveri_DSM555_GlcD (CKL_RS17120), M_elsdenii_DSM20460 (MELS_RS10260), and M_elsdenii_DSM20460_acyl-CoA (MELS_RS10965).

The *C. diolis* NJP7 EtfB: CCS79_RS24285 is truncated at the N-terminus being found at the edge of the contig. The *C. diolis* NJP7 EtfA: CCS79_RS18965 and EtfBs: CCS79_RS25810 and CCS79_RS25815 were omitted from the analysis.

Three sets of proteins were subjected to phylogenetic analysis: the EtfA and EtfB proteins, and their concatenated counterparts prepared with a custom Python script. Protein sets were aligned using ClustalW [30] with default parameters (Cost matrix: ID, Gap open cost: 8, Gap extend cost: 0.1). Phylogenetic trees were built using Geneious Tree Builder with the following parameters: Genetic Distance Model: Jukes-Cantor [31], Tree build Method: UPGMA [32], Resampling Method: bootstrap, Number of replicates: 1000, Support Threshold: 30%.

## Results and discussion

### Transformation of lactate and acetate to butyrate in batch experiments

A critical review of studies on hydrogen production during the acidic step of anaerobic digestion led us to postulate that a phenomenon analogous to cross-feeding of lactate in the gastrointestinal tract occurs in dark fermentation bioreactors [4, 8–10, 12, 33–39]. Our previous examination of hydrogen-yielding microbial communities in packed-bed reactors supplied with media containing molasses in a continuous system revealed that despite the major contribution of lactic acid bacteria, there is no net production of lactate, and butyrate is the main metabolite [23].

**Fig. 1:**
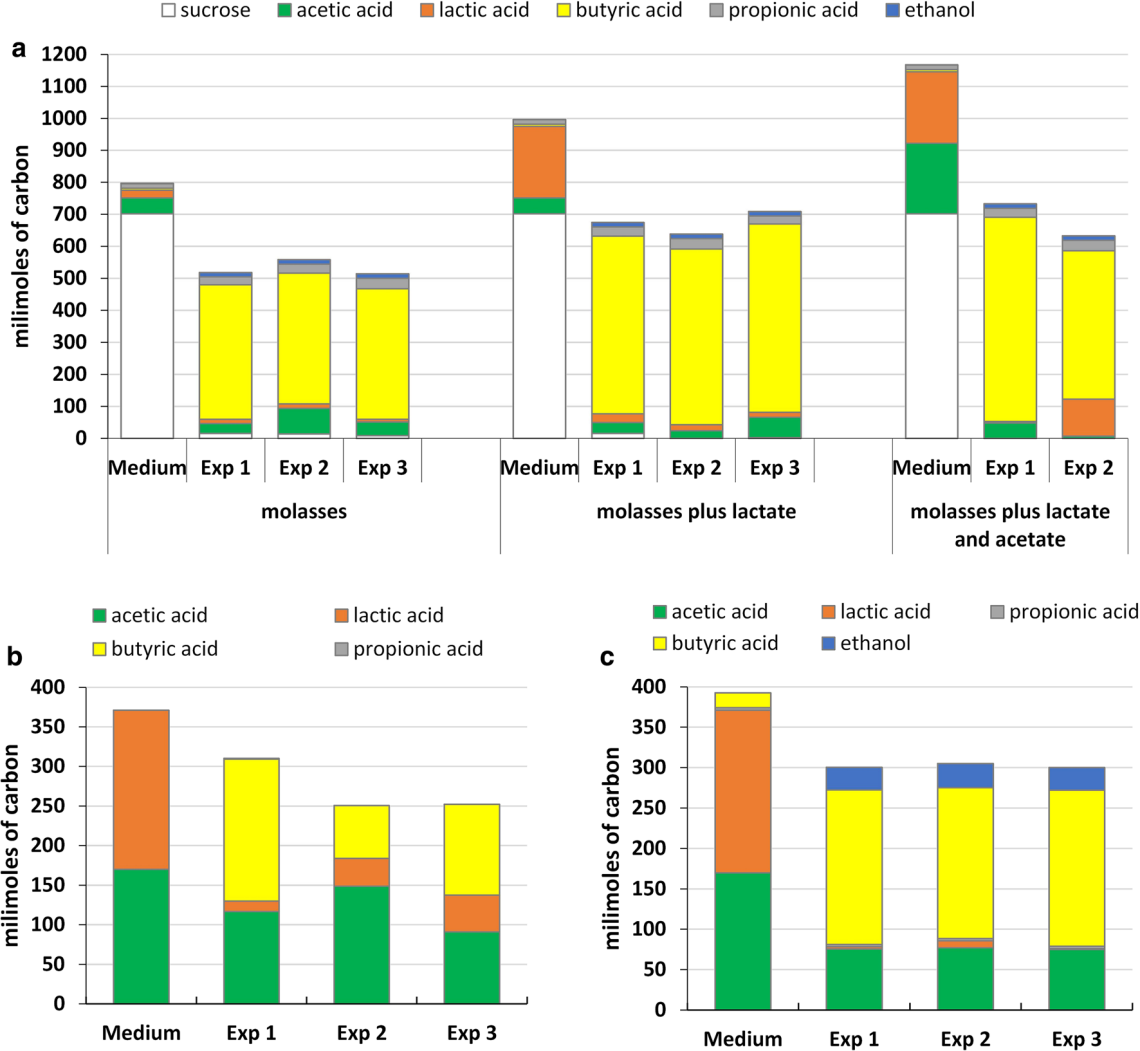
Non-gaseous fermentation products shown in millimoles of carbon in batch cultures of microbial community from a dark fermentation bioreactor (**a**, **b**) or *C. butyricum* (**c**), processing medium containing molasses enriched with lactate and acetate (**a**) or lactate and acetate medium without carbohydrates (**b**, **c**). The data come from two parallel measurements

Here, we present the results of three series of batch experiments focused on the conversion of lactate and acetate to butyrate by microbial community from dark fermentation bioreactor and a pure culture of *C. butyricum*, summarized in Table 1. Table 1 also shows the density of bacterial cultures measured by OD_600nm_ and the pH inside the flasks during fermentation process. Figure 1 presents composition of cultivation media and non-gaseous fermentation products in millimoles of carbon.

Butyrate is a typical product of hydrogen-yielding saccharolytic clostridial-type fermentation. Thus, butyrate was an abundant non-gaseous fermentation product when molasses was a component of the medium processed by the microbial community from dark fermentation bioreactors in the first series of experiments. Lactate was also found as a fermentation product. It is noteworthy that lactate, butyrate and acetate were also detected as components of the starting molasses-containing medium (Fig. 1a). When the molasses-containing medium was supplemented with additional lactate or lactate and acetate, the lactate was utilized by the microbial communities in 88–98%, and in one case (the “molasses plus lactate and acetate” experiment 2) in 48% (Fig. 1a).

In the next experimental approach, the medium contained only sodium lactate and sodium acetate as carbon sources. The 77–94% of lactate was used by microbial communities. The main components of the post-culture fluids were butyrate and acetate (Fig. 1b). These results are in agreement with those of previous studies [8, 9, 11]. It should be noted that acetate is a substrate and an intermediate on the pathway of lactate to butyrate transformation [1, 5, 6]. Interestingly, in all the tested variants the additional lactate did not affect the generally very low concentration of propionate within the non-gaseous fermentation products. This indicates the absence of any propionate-type fermentation characteristic of e.g. *Clostridium propionicum* [8] in these batch cultures.

The final series of experiments examined the growth of a pure culture of *C. butyricum* on medium containing lactate and acetate supplemented with yeast extract. The results showed for the first time that, in the absence of carbohydrates, *C. butyricum*, similarly to other representatives of the *Firmicutes* (*C. acetobutylicum* [5], *Butyribacterium methylotrophicum* [6], *C. diolis* [7]), utilizes lactate and acetate, and converts them to butyrate. The experiment lasted for 9 days till the bacterial culture achieved the optical density OD_600nm_ ≈ 0.7 (Table 1). After that time the optical density of the culture decreased. Microscopic observation revealed (data not shown) that during the experiment part of the cells formed endospores. On average 98% of lactate was utilized by *C. butyricum* and a significant increase of butyrate was detected (Fig. 1c). It should be noted that the yeast extract was also a source of butyrate (4.6 mM) and propionate (1 mM) in the medium. No increase in propionate concentration was observed in the culture, while ethanol was an additional product of bacterial metabolism. The presence of ethanol was not determined in previous studies on butyrate production from acetate and lactate by pure strains [5–7]. It is noteworthy that no growth of *C. butyricum* was observed when the medium contained lactate as a sole carbon source indicating that (i) both lactate and acetate are required for bacterial growth; (ii) lactate cannot be transformed to propionate as in the case of *C. propionicum* [8].

The approximate balance of carbon in millimoles for the *C. butyricum* experiments was based on the following reasoning involving concentration of acetate, lactate, propionate, butyrate and ethanol in the medium and the post-cultured fluids:

1 $$ \begin{aligned} & 170\, acetate + 200\, lactate + 3\, propionate + 18\, butyrate \hfill \\ &  \quad \quad \to 76 \,acetate + 4 \,lactate + 3 \, propionate + 190\, butyrate + 29 \,ethanol + X \hfill \\ \end{aligned} $$

where X was the estimated bacterial biomass and other products as fermentation gases (carbon dioxide).

It was assumed that the excess of acetate in the medium and the yeast extract-derived butyrate and propionate were not metabolized, thus the approximate balance of carbon was as follows:

2 $$ 100\, acetate + 200 \,lactate \to 170 \,butyrate + 30 \,ethanol + X $$

where X is bacterial biomass and other fermentation products (estimated as 100 millimoles C).

The fermentation balance was further used for the proposed scheme of lactate and acetate conversion to butyrate in *C. butyricum*; see the section on the enzymatic machinery of lactate and acetate transformation to butyrate.

### The identification of *etfA/B* genes and their neighbourhood in the *C. butyricum* genome

**Fig. 2:**
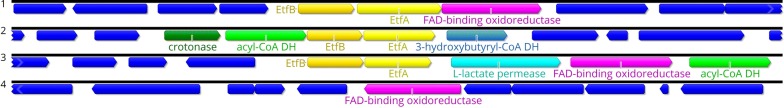
Four fragments of the NZ_CP013252 chromosome of *C. butyricum* KNU-L09 containing genes encoding EtfA (1: ATN24_RS03030, 2: ATN24_RS03165, 3: ATN24_RS08885) and EtfB (1: ATN24_RS03025, 2: ATN24_RS03160, 3: ATN24_RS08880), and potentially GlcD (1: ATN24_RS03035, 3: ATN24_RS08895, 4: ATN24_RS11095)

The mechanism of transformation of lactate and acetate to butyrate proposed for gastrointestinal tract bacteria [1] and bacteria conducting butyric acid fermentation [5, 6] was demonstrated before the discovery of the flavin-based electron bifurcation mechanism. Since *C. butyricum* is able to convert lactate and acetate to butyrate we selected the genome of *C. butyricum* KNU-L09 (completed genome) for the presence of sequences encoding EtfAB complexes. BLAST searches revealed the existence of three gene clusters for EtfA/B complexes in the genome of *C. butyricum* KNU-L09 (Fig. 2), all within the chromosome NZ_CP013252. One of them (named 2 in Fig. 2) comprises acyl-CoA dehydrogenase and two 3-hydroxybutyryl-CoA dehydrogenases (one annotated as crotonase). The other two (named 1 and 3 in Fig. 2) contain FAD-binding oxidoreductase (homologous to lactate dehydrogenase GlcD of *A. woodii*), and l-lactate permease and acyl-CoA dehydrogenase, respectively. The *C. butyricum* KNU-L09 genome encodes one other FAD-binding oxidoreductase with potential lactate dehydrogenase activity, denoted as cluster 4 in Fig. 2.

**Fig. 3:**
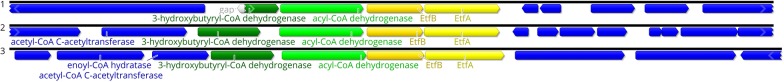
Fragments of the chromosomes of *Roseburia intestinalis* L1-82 (1), *Eubacterium rectale* ATCC 33656 (2) and *Faecalibacterium prausnitzii* A2165 (3) showing the EtfA (1: ROSINTL182_RS00140, 2: EUBREC_RS03305, 3: CG447_RS01775) and EtfB (1: ROSINTL182_RS00145, 2: EUBREC_RS03300, 3: CG447_RS01780) genes and their genetic contexts

A similar search was performed for the selected genomes of bacteria *Roseburia intestinalis* L1-82, *Eubacterium rectale* ATCC 33656, and *Faecalibacterium prausnitzii* A2165 recognized as butyrate producers but incapable of lactate oxidation [1, 28]. As a result, only one cluster containing *etfA* and *etfB* genes with acyl-CoA and butyryl-CoA dehydrogenases encoding genes was found (Fig. 3). An additional search for l-lactate permease in these species was performed. No genes encoding l-lactate permease was identified in these genomes.

### Phylogenetic relationship of EtfAs and EtfBs from selected species capable of lactate oxidation

**Fig. 4:**
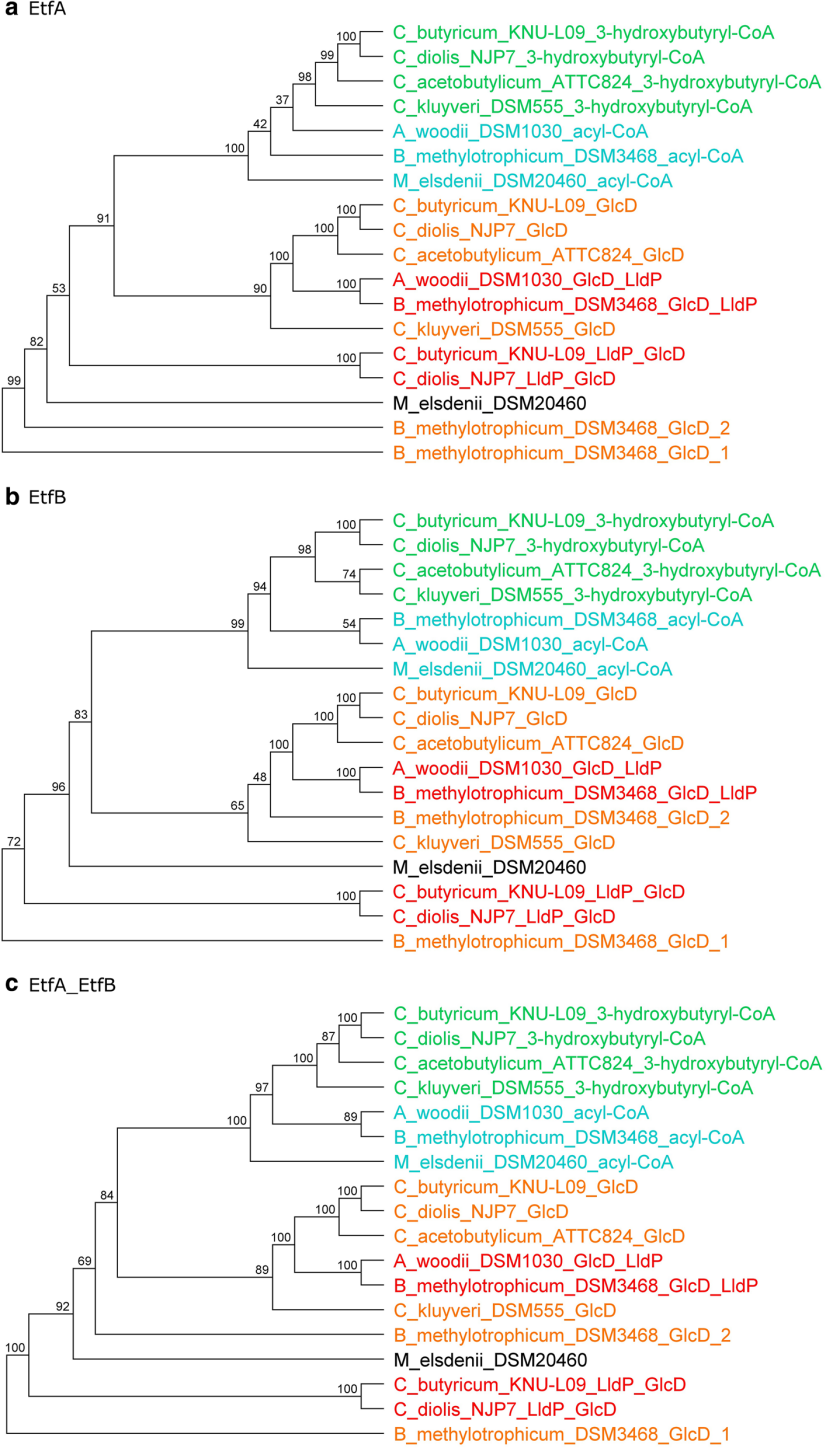
Phylogenetic trees of EtfA (**a**), EtfB (**b**), and concatenated EtfA_EtfB (**c**) proteins for selected species (see Materials and Methods). After the name of species there is a shortcut for the relevant enzyme coded in the vicinity of *etfA/B*: _GlcD—in the vicinity of FAD/FMN-containing dehydrogenase, probable D-lactate dehydrogenase (genetic context arrangement similar to fragment 1 in Fig. 2); _3-hydroxybutyryl-CoA—in the vicinity of 3-hydroxybutyryl-CoA dehydrogenase (genetic context arrangement similar to fragment 2 in Fig. 2); _LldP_GlcD—in the vicinity of l-lactate permease and FAD/FMN-containing dehydrogenase, probable d-lactate dehydrogenase (genetic context arrangement similar to fragment 3 in Fig. 2); _acyl-CoA—in the vicinity of acyl-CoA dehydrogenase

A phylogenetic analysis of the EtfAs and EtfBs proteins from several species was performed. All of the selected bacteria are recognized lactate oxidisers that are either unable to (*Acetobacterium woodii*) or able to synthesize butyrate (*Butyribacterium methylotrophicum*, *Clostridium acetobutylicum, Clostridium butyricum, Clostridium diolis*, *Clostridium kluyveri*, *Megasphaera elsdenii*). The genomes of the analysed species capable of transforming lactate into butyrate encode at least two different EtfA/EtfB proteins and these genes are found in different genetic contexts (data not shown), similarly to those shown for *C butyricum* in Fig. 2, i.e. in the vicinity of genes encoding (i) l-lactate permease and lactate oxidase, or (ii) 3-hydroxybutyryl-CoA dehydrogenase. As shown in Fig. 4, the EtfA and EtfB proteins encoded by genes associated with a 3-hydroxybutyryl-CoA dehydrogenase gene form a distinct group, with bootstrap support of 98–100%. This group is related to the Etf proteins encoded by genes associated with acyl-CoA dehydrogenase genes. On the other hand, the EtfA/EtfB proteins encoded by genes in the context of GlcD- and/or LldP-encoding genes form a separate cluster, with bootstrap support of at least 65%. Only the *C. butyricum* KNU-L09 EtfA/B proteins with coding sequences in the vicinity of LldP and GlcD genes, and those of *B. methylotrophicum* DSM3468 encoded in the vicinity of the GlcD gene, appear to form outgroups. Topologies with the same tendencies were obtained for trees of the EtfA only, EtfB only, and concatenated EtfA and EtfB proteins (Fig. 4).

There is more extent similarity between Etf subunits that catalyse the same reactions in various species than between the different *etf* gene products within the same species. This indicates that Etf complexes are reaction-specific. Further experiments using clostridial *etf* mutants are required to confirm this notion.

Our results are in agreement with those of Garcia Costas [22]. The Etfs analysed in our study belong exclusively to group G2; the EtfA and EtfB proteins encoded by genes associated with a 3-hydroxybutyryl-CoA and acyl-CoA dehydrogenase genes to subgroup G2A involved in butyrate metabolism whereas the EtfA/EtfB proteins encoded by genes in the context of GlcD- and/or LldP-encoding genes to subgroup G2B involved in lactate metabolism. The presented here phylogenetic analysis is limited to the bacteria able to oxidise lactate and form butyrate. It contributes to explanation of cross-feeding of lactate, nutritional interaction between lactate- and acetate-forming bacteria and butyrate producers in different environments such as the human colon or dark fermentation bioreactors, on molecular level.

### Enzymatic machinery of lactate and acetate transformation to butyrate

**Fig. 5:**
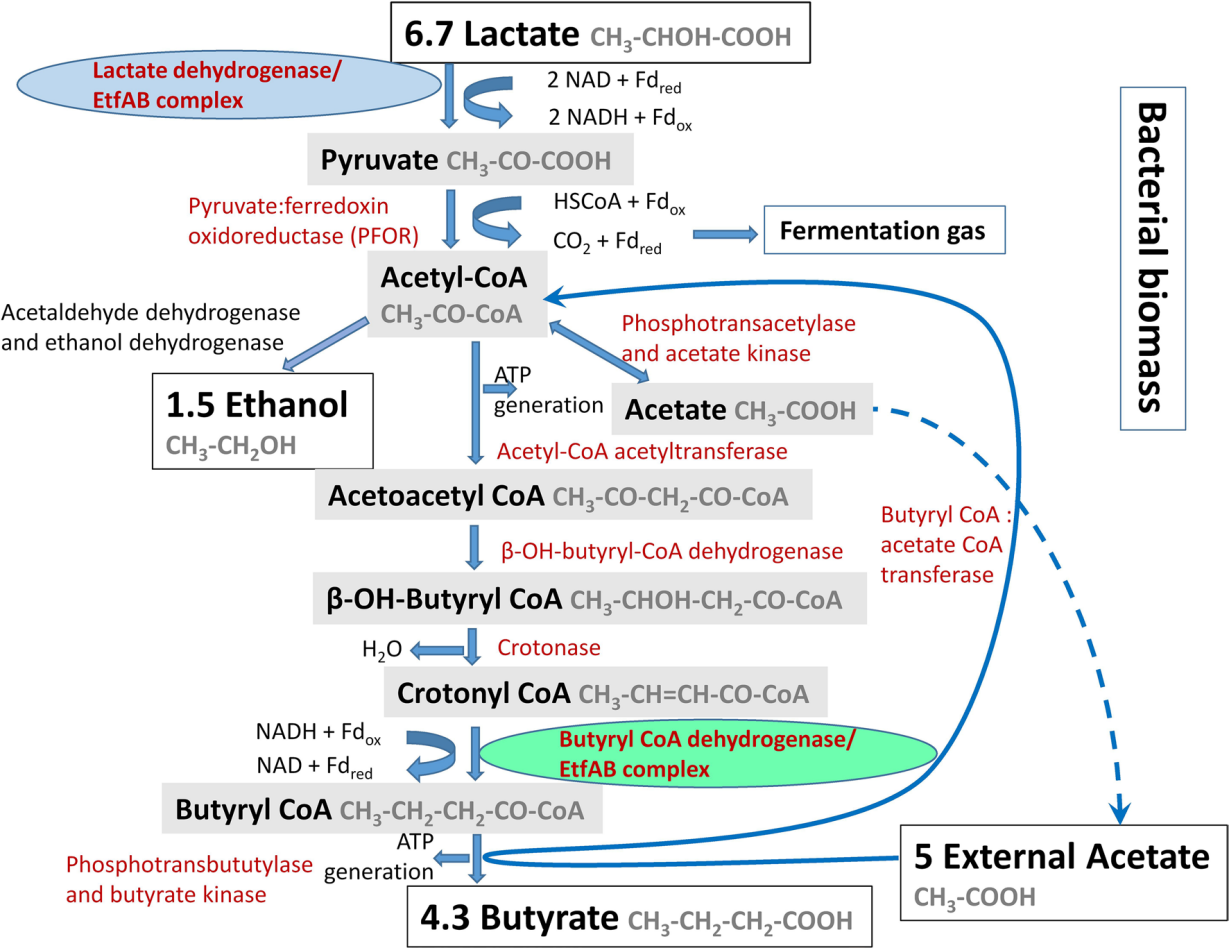
A proposed scheme of the metabolic pathway of lactate and acetate transformation to butyrate and other products by *C. butyricum* based on carbon balance presented by the Eq. 2. The millimoles of carbon were converted to molecules of acetate, lactate, butyrate, ethanol, and the obtained values were multiplied by 100. The scheme presents the results of this study in relation to the pathway of lactate and acetate conversion to butyrate in *Firmicutes* [1, 5, 6] as well as current knowledge on anaerobic lactate oxidation and butyryl-CoA formation including flavin-based electron bifurcation [14–20]

After considering the above results in relation to the common scheme of lactate and acetate conversion to butyrate in *Firmicutes* [1, 5, 6] and current knowledge on flavin-based electron bifurcation [14, 18, 20], we propose an updated metabolic scheme on the example of *C. butyricum* (Fig. 5). This scheme involves the contribution of two different EtfAB complexes: the lactate dehydrogenase- and crotonylCoA dehydrogenase-specific forms. The activities of these complexes may probably constitute the X factor described in previous studies [5, 6]. Notice that it is only a simplified scheme including possible reactions that can be modified by operational conditions, bacterial growth phase, metabolite concentration.

Briefly, a FAD-dependent lactate dehydrogenase LDH, in a stable complex with an electron transfer flavoprotein (EtfA/B), catalyzes endergonic lactate oxidation using NAD^+^ as the oxidant, which is accompanied by the simultaneous oxidation of reduced ferredoxin. The subsequent steps are analogous to those of butyric acid fermentation (saccharolytic clostridial-type fermentation) [17]. Pyruvate is oxidized to acetyl coenzyme A (acetyl-CoA), which is further routed to acetate and butyrate. Acetate is produced via acetate kinase in a pathway generating energy in the form of ATP. For butyrate formation, two molecules of acetyl-CoA are condensed to form one molecule of acetoacetyl-CoA, and this is then reduced to butyryl-CoA. The final step requires a butyryl-CoA dehydrogenase/EtfAB complex catalyzing endergonic ferredoxin reduction with NADH coupled to exergonic crotonyl-CoA reduction with NADH. Butyrate can be synthesized via two metabolic pathways: (i) phosphotransbutyrylase and butyrate kinase, and (ii) butyryl CoA:acetate CoA transferase. Butyryl-CoA:acetate CoA-transferase transports the CoA component to external acetate, resulting in the release of butyrate and acetyl-CoA. Acetyl-CoA can be transformed to ethanol by acetaldehyde dehydrogenase and ethanol dehydrogenase. Ethanol synthesis in the context of lactate and acetate transformation to butyrate has not been considered in previous studies [1, 2, 5–7, 40]. Formation of other fermentation products and bacterial biomass production were also noted in the scheme (Fig. 5).

The findings of this study have increased our understanding of metabolic pathways and the symbiotic relationships between bacteria during acidogenesis.

## Conclusions

The results of this study have confirmed that lactate and acetate is converted to butyrate by microbial communities from dark fermentation bioreactors. *C. butyricum* was used as a new model to study the transformation of lactate and acetate to butyrate. Notably, ethanol was found among the non-gaseous fermentation products. The identification of *etfA/B* genes in the genomes of *C. butyricum* and other species capable of lactate and acetate to butyrate conversion indicates the reaction-specificity of different Etf complexes. Finally, we propose a metabolic pathway of lactate and acetate transformation by *C. butyricum*.

## Abbreviations

Etf: electron transfer flavoprotein
FAD: flavin adenine dinucleotide
GlcD: domain of FAD-dependent lactate dehydrogenase, FAD/FMN-containing dehydrogenase WP_014355267 (AWO_RS04420)
HPLC: high performance liquid chromatography
LDH: lactate dehydrogenase
LldP: lactate permease
OD: optical density
